# Comparative analysis and phylogenetic investigation of Hong Kong Ilex chloroplast genomes

**DOI:** 10.1038/s41598-021-84705-9

**Published:** 2021-03-04

**Authors:** Bobby Lim-Ho Kong, Hyun-Seung Park, Tai-Wai David Lau, Zhixiu Lin, Tae-Jin Yang, Pang-Chui Shaw

**Affiliations:** 1 Li Dak Sum Yip Yio Chin R & D Centre for Chinese Medicine and Institute of Chinese Medicine, The Chinese University of Hong Kong, ShatinHong Kong, N.T. China; 2 Department of Agriculture, Forestry and Bioresources, Plant Genomics & Breeding Institute, College of Agriculture & Life Sciences, Seoul National University, Seoul, Republic of Korea; 3 Shiu-Ying Hu Herbarium, School of Life Sciences, The Chinese University of Hong Kong, ShatinHong Kong, N.T. China; 4 School of Chinese Medicine, The Chinese University of Hong Kong, ShatinHong Kong, N.T China

**Keywords:** Genome, Plant sciences

## Abstract

*Ilex* is a monogeneric plant group (containing approximately 600 species) in the Aquifoliaceae family and one of the most commonly used medicinal herbs. However, its taxonomy and phylogenetic relationships at the species level are debatable. Herein, we obtained the complete chloroplast genomes of all 19 *Ilex* types that are native to Hong Kong. The genomes are conserved in structure, gene content and arrangement. The chloroplast genomes range in size from 157,119 bp in *Ilex graciliflora* to 158,020 bp in *Ilex kwangtungensis*. All these genomes contain 125 genes, of which 88 are protein-coding and 37 are tRNA genes. Four highly varied sequences (*rps16-trnQ, rpl32-trnL, ndhD-psaC* and *ycf1*) were found. The number of repeats in the *Ilex* genomes is mostly conserved, but the number of repeating motifs varies. The phylogenetic relationship among the 19 *Ilex* genomes, together with eight other available genomes in other studies, was investigated. Most of the species could be correctly assigned to the section or even series level, consistent with previous taxonomy, except *Ilex rotunda* var. *microcarpa, Ilex asprella* var. *tapuensis* and *Ilex chapaensis*. These species were reclassified; *I. rotunda* was placed in the section *Micrococca*, while the other two were grouped with the section *Pseudoaquifolium*. These studies provide a better understanding of *Ilex* phylogeny and refine its classification.

## Introduction

*Ilex*, a monogeneric plant group in the family Aquifoliaceae, is a widespread genus. It can either be evergreen or deciduous and can be found throughout subtropical regions. There are approximately 600 species in total, and some of them have medical uses^1^. Some kinds of *Ilex* are commonly used herbs in traditional Chinese medicine (TCM), and they are effective in treating influenza, relieving pain and anti-inflammation^2,3^. The mechanism has been recently proposed. Asprellcosides from *I. asprella* can significantly inhibit the replication of influenza A virus, while rotundarpene from *I. rotunda* var. *microcarpa* can inhibit the TNF-α-mediated pathway^4,5^.

*Ilex* species have a variety of pharmacological properties, and accurate classification of them is needed. Cuénoud and colleagues used the *rbcL* and *atp-rbcL* spacer to study the phylogenetic relationship of *Ilex.* A total of 116 *Ilex* species were classified into the American clade, Asian/North American clade, deciduous clade and Eurasian clade^6^. Manen and colleagues used both chloroplast markers and the 5S RNA spacer to assign 105 *Ilex* into the 4 clades. After that, *Ilex* was further assigned to different subgenera, sections and alliances^7^. Nuclear DNA ITS and *nepGS* were also included in recent research^1^. In addition to the commonly used DNA regions, Lin and colleagues have also used the nuclear segment *gapC* to study the speciation of *Ilex*^8^. According to *Flora of China, Ilex* can be divided into three subgenera, Byronia, Ilex and Prinos^9^. These three subgenera can be further classified into taxonomic sections and series. The classification was generally based on morphological analysis, and only short universal DNA markers of some species were included in the studies.

Chloroplasts are responsible for multiple functions, such as photosynthesis^10^, amino acid synthesis and nitrogen metabolism in plants^11,12^. As most DNA markers, such as *rbcL*, *matK* and *psbA*, are located in the chloroplast genome, the development of chloroplast whole-genome sequencing has contributed to solving the phylogenetic relationship of plants^13–15^. In addition to classical DNA barcoding regions, novel DNA markers can also be found^16^.

Here, we obtained the complete chloroplast genomes of 19 *Ilex* species native to Hong Kong. Divergence hotspot investigation, repeat analysis and polymorphism studies were performed. Through including other published *Ilex* chloroplast genomes, topologies of the *Ilex* phylogenies were constructed.

## Results

### Chloroplast genome assembly

**Figure 1:**
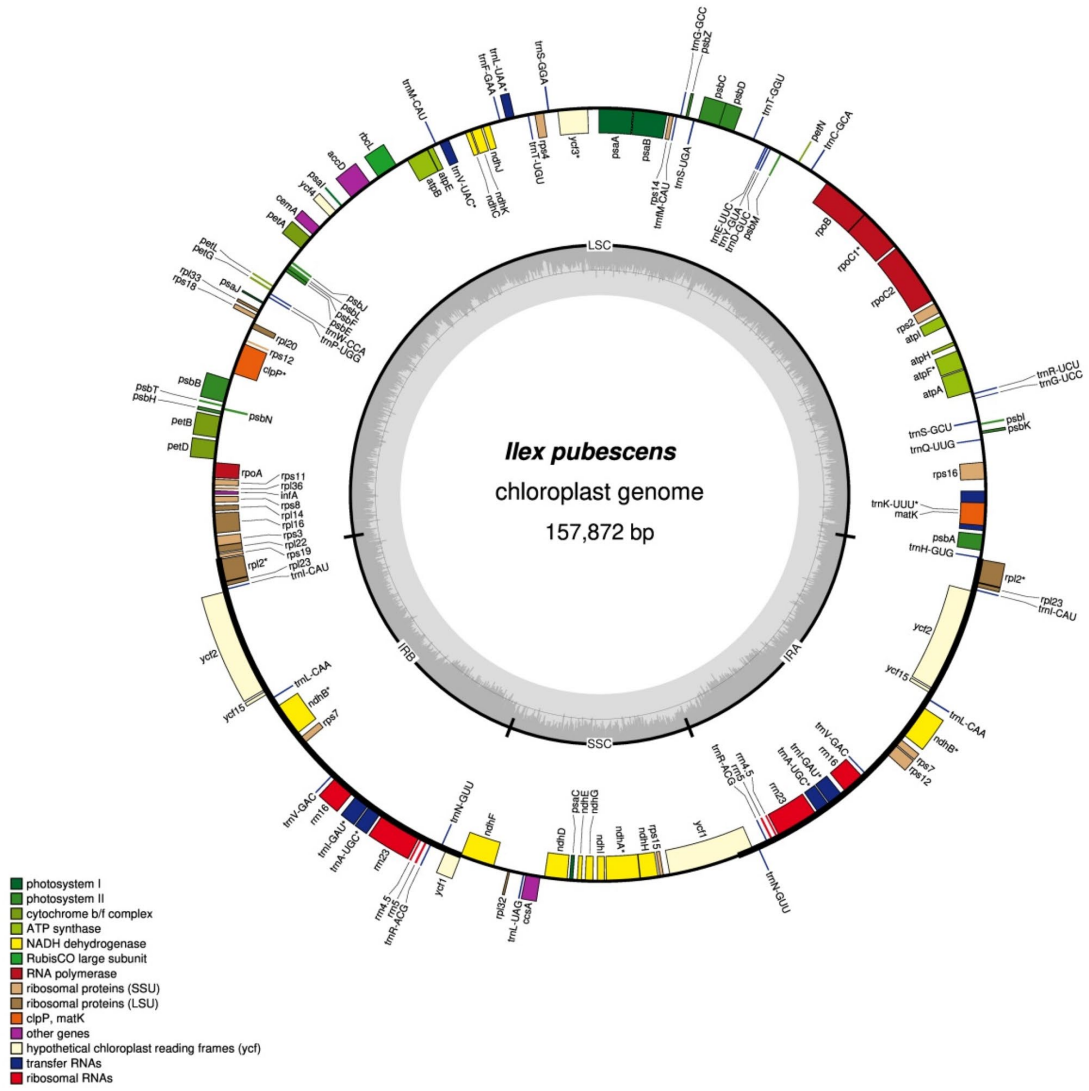
Chloroplast genome map of *I. pubescens.* The genome structure and gene arrangement in the 19 *Ilex* species were conserved. Introns containing coding regions are indicated with asterisks. The inner grey circle represents the GC content of each region. Genes shown in the inner circle are transcribed clockwise and vice versa. Genes belonging to different functional groups are indicated in different colours.

By using the Illumina NovaSeq 6000 system, the complete chloroplast genomes of 19 *Ilex* species were sequenced. Raw data were generated with an average read length of 150 bp. Complete chloroplast sequences were assembled by de novo assembly and validated by mapping the raw reads into the assembled contigs. Sequences of the reads and contigs were compared, and the final plastomes were submitted to GenBank with accession numbers according to the supplementary material (Supplementary Table S1). After that, 19 *Ilex* genomes were used for indel, SSR and REPuter analyses. The corresponding genome map of *I. pubescens* is shown as a reference (Fig. 1).

### Genome structure and gene content

These genomes followed the typical quadripartite structure of angiosperms, which consisted of one LSC Sect. (86,506–87,400 bp), one SSC Sect. (18,380–18,442 bp) and a pair of IR regions (26,065–26,125 bp). The GC content ranged from 37.6% to 37.7%, which was consistent with a previous report of *Ilex* chloroplast genomes^17–19^. All of the 19 *Ilex* species have the same gene content and gene order. They contain 125 genes, of which 88 are protein-coding genes, 37 are tRNA genes and 8 are encode rRNA (Table 1). Seventeen genes contain one or more introns, of which 12 (*ycf3, atpF, petB, petD, ndhA, ndhB, rpoC1, rps12, rps16, rpl2, rpl16,* and *clpP*) are protein-coding, and five are responsible for tRNA (*trnA*-UGC, *trnI*-GAU, *trnL*-UAA, *trnQ*-UUG, and *trnV*-UAC). Several genes are duplicated in the IR regions, among which 8 (*ndhB, rps7, rps12, rpl2, rpl23, ycf1, ycf2,* and *ycf15*) are protein coding, 7 (*trnA*-UGC, *trnI-*CAU, *trnI*-GAU, *trnL*-CAA, *trnN-*GUU, *trnR*-ACG, and *trnV*-GAC) encoded tRNAs, and 4 (rrn4.5, rrn5, rrn16, and rrn23) encode rRNA (Table 2). Only one pseudogene, *ycf1*, was found in all *Ilex* genomes. This observation was consistent with the seven *Ilex* genomes in Yao’s study^17^. The similarities in the genome structure and genetic content indicate that the chloroplast genomes of all 19 *Ilex* species are highly conserved.

**Table 1:** Summary of the assembly data for *Ilex* chloroplast genomes.

Species	Genome size (bp)	LSC (bp)	IR (bp)	SSC (bp)	Number of genes	rRNA	tRNA	Protein-coding genes	A%	C%	G%	T%	GC%
*I. latifolia*	157,558	86,945	26,093	18,427	125	8	37	88	30.9	19.1	18.5	31.5	37.6
*I. lohfauensis*	157,469	86,873	26,078	18,440	125	8	37	88	30.8	19.2	18.5	31.5	37.6
*I. kwangtungensis*	158,020	87,400	26,104	18,412	125	8	37	88	30.9	19.1	18.5	31.5	37.6
*I. triflora*	157,706	87,183	26,065	18,393	125	8	37	88	30.8	19.2	18.5	31.5	37.7
*I. ficoidea*	157,536	86,922	26,094	18,426	125	8	37	88	30.8	19.2	18.5	31.5	37.6
*I. rotunda* var. *microcarpa*	157,780	87,094	26,125	18,436	125	8	37	88	30.8	19.2	18.5	31.5	37.6
*I. asprella*	157,856	87,265	26,075	18,441	125	8	37	88	30.8	19.1	18.5	31.6	37.6
*I. pubescens*	157,872	87,285	26,073	18,441	125	8	37	88	30.8	19.1	18.5	31.6	37.6
*I. asprella* var. *tapuensis*	157,671	87,161	26,065	18,380	125	8	37	88	30.8	19.2	18.5	31.5	37.7
*I. hanceana*	157,478	86,889	26,074	18,441	125	8	37	88	30.8	19.2	18.5	31.5	37.6
*I. cinerea*	157,215	86,601	26,094	18,426	125	8	37	88	30.8	19.2	18.5	31.5	37.7
*I. championii*	157,468	86,878	26,074	18,442	125	8	37	88	30.8	19.2	18.5	31.5	37.6
*I. graciliflora*	157,119	86,506	26,093	18,427	125	8	37	88	30.8	19.2	18.5	31.5	37.7
*I. memecylifolia*	157,842	87,249	26,076	18,441	125	8	37	88	30.8	19.1	18.5	31.6	37.6
*I. chapaensis*	157,665	87,155	26,065	18,380	125	8	37	88	30.8	19.2	18.5	31.5	37.7
*I. cornuta*	157,216	86,607	26,091	18,427	125	8	37	88	30.8	19.2	18.5	31.5	37.7
*I. lancilimba*	157,998	87,382	26,105	18,406	125	8	37	88	30.9	19.2	18.5	31.5	37.6
*I. dasyphylla*	158,009	87,388	26,105	18,411	125	8	37	88	30.9	19.1	18.5	31.5	37.6
*I. viridis*	157,661	87,147	26,065	18,384	125	8	37	88	30.8	19.2	18.5	31.5	37.7

**Table 2:** Gene content and functional classification of the 19 *Ilex* chloroplast genomes.

Gene category	Gene function	Gene name
Photosynthesis-related genes	Rubisco	*rbcL*
	Photosystem I	*psaA, psaB, psaC, psaI, psaJ*
	Assembly/stability of photosystem I	**ycf3, ycf4
	Photosystem II	*psbA, psbB, psbC, psbD, psbE, psbF, psbH, psbI, psbJ, psbK, psbL, psbM, psbN, psbT, psbZ*
	ATP synthase	atpA, atpB, atpE, *atpF, atpH, atpI
	Cytochrome b/f complex	petA, *petB, *petD, petG, petL, petN
	Cytochrome c synthesis	*ccsA*
	NADPH dehydrogenase	*ndhA, *ndhB(× 2*), ndhC, ndhD, ndhE, ndhF, ndhG, ndhH, ndhI, ndhJ, ndhK*
Transcription- and translation-related genes	Transcription	rpoA, rpoB, *rpoC1, rpoC2,
	Ribosomal protein	*rps2, rps3, rps4, rps7(*× 2), rps8, rps11, *rps12(× 2), rps14, rps15, *rps16, rps18, rps19, *rpl2(× 2), rpl14, *rpl16, rpl20, rpl22, rpl23(× *2), rpl32, rpl33, rpl36*
	Translation initiation factor	*infA*
RNA genes	Ribosomal RNA	rrn4.5(× 2), rrn5(× 2), rrn16(× 2), rrn23(× 2)
	Transfer RNA	*trnA-UGC(× 2), *trnC*-GCA, *trnD*-GUC, *trnE*-UUC, *trnF*-GAA, *trnG*-GCC, *trnG*-UCC, *trnH*-GUG, *trnI*-CAU(× 2), *trnI-GAU(× 2), *trnK*-UUU, *trnL*-CAA(× 2), *trnL-UAA, *trnL*-UAG, *trnM*-CAU, *trnfM*-CAU, *trnN*-GUU(× 2), *trnP*-UGG, *trnQ-UUG, *trnR*-ACG(× 2), *trnR*-UCU, *trnS*-GGA, *trnS*-GCU, *trnS*-UGA, *trnT*-UGU, *trnT*-GGU, *trnV*-GAC(× 2), *trnV-UAC, *trnW*-CCA, *trnY*-GUA
Miscellaneous group	Maturase	*matK*
	Inner membrane protein	*cemA*
	ATP-dependent protease	**clpP
	Acetyl-CoA carboxylase	*accD*
	Unknown functions	*ycf1*(× 2), *ycf2*(× 2), *ycf15*(× 2)

Intron-containing genes are labelled with asterisks. The number of introns corresponds to the number of asterisks.

### Simple and complex repeats analysis

**Figure 2:**
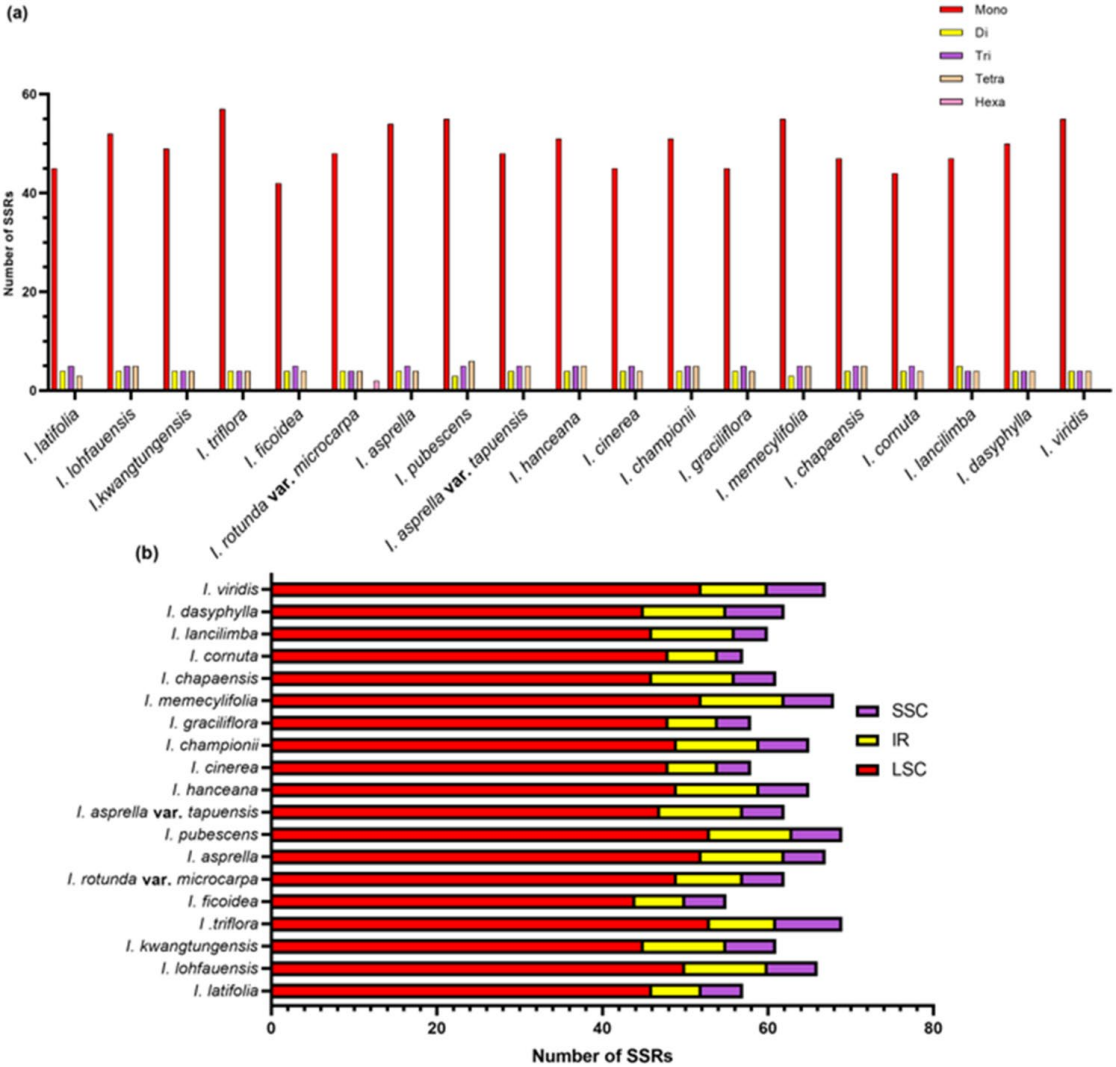
Classification of simple sequence repeats (SSRs) in the chloroplast genomes of 19 *Ilex* species based on **(a)** repeat motifs of SSRs and **(b)** the distribution of SSRs.

Simple sequence repeats (SSRs) are repeating DNA motifs that range from one to six nucleotides^20^. The number and position of repeated motifs are thought to be useful in population genetic studies and have been widely used in finding polymorphisms in chloroplast genomes^21,22^. By using MISA analysis tools, the number and position of different SSR motifs in 19 *Ilex* were investigated. All of the *Ilex* species do not have penta-nucleotide motifs, while *Ilex rotunda* var. *microcarpa* is the only species containing hexa-nucleotide repeat motifs. Mono-nucleotide repeat motifs account for the largest proportion, ranging from 44 to 56 (Fig. 2a). Most of the mono-nucleotide repeat motifs are A/T repeats, while only 5 species, *I. triflora, I. viridis, I. asprella* var. *tapuensis, I. cinerea* and *I. graciliflora*, have C/G repeat motifs. A/T repeats occupy at least 75.9% of the total SSR, and the phenomenon of AT richness in the SSR of terrestrial plants has been reported in previous studies^23,24^. The numbers and types of di-, tri- and tetra-nucleotide motifs are mostly conserved among *Ilex*, except for the ACAT/ATGT tetra-nucleotide motif, which is present only in *I. ficoidea* and *I. pubescens* (Supplementary Table S2). Regarding the SSR distribution, most of the SSRs are found in the LSC and SSC regions but not the IR regions, which is consistent with studies in angiosperms (Fig. 2b)^25–27^. Owing to the variation in the LSC, IR and SSC lengths, densities of SSRs in different regions were also calculated (Supplementary Table S3). Only a few SSRs were found in the two IR regions; therefore, only slight variation was observed in these regions. On the other hand, relatively large variation in the SSR density was noticed in the SSC regions, which varied from 1.63E−04 in *I. cornuta* to 4.35E-04 in *I. triflora*.

**Figure 3:**
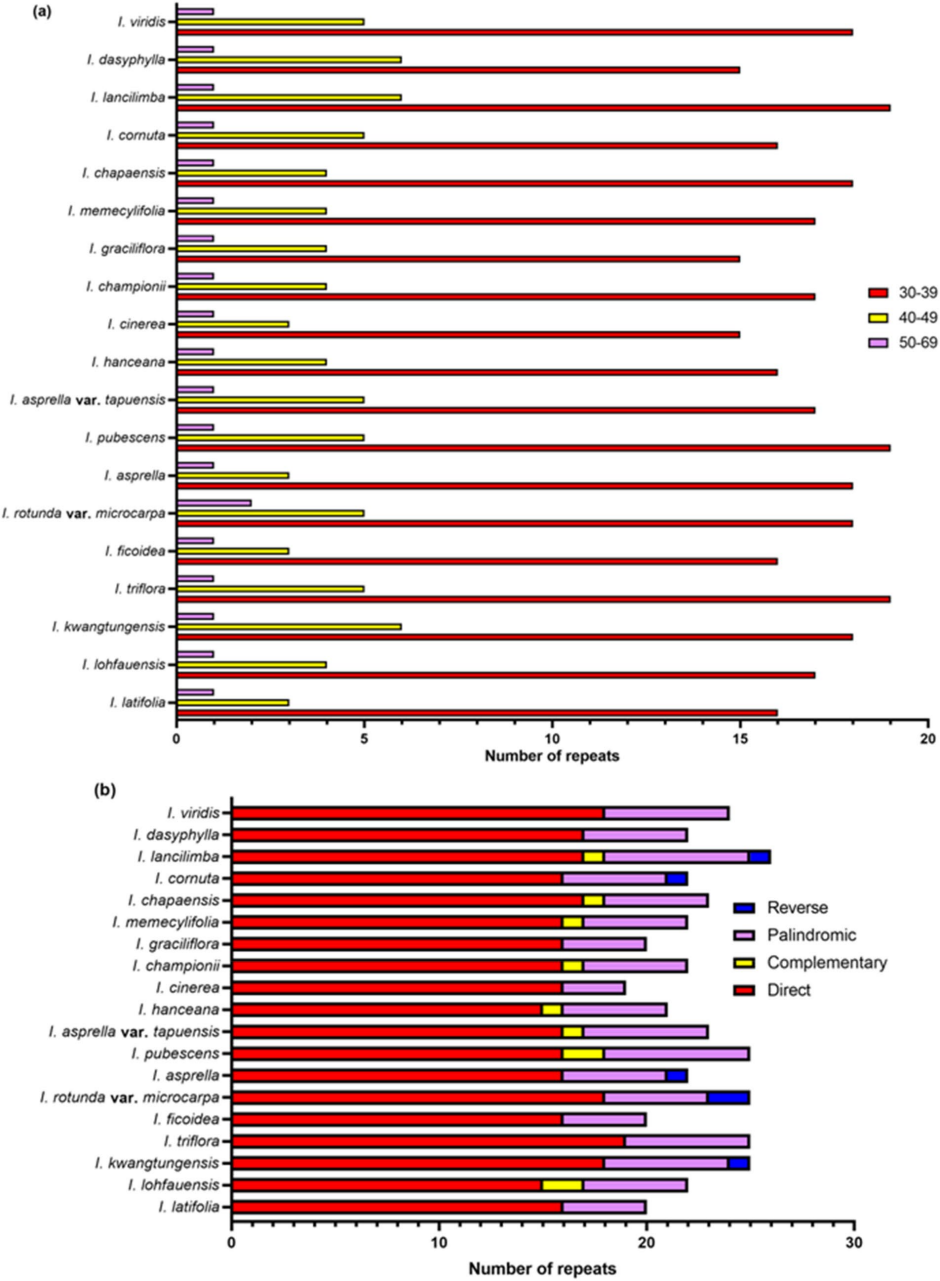
**(a)** Length of repeats and **(b)** frequency of repeating motifs of the 19 *Ilex* chloroplast genomes determined by REPuter.

Complex repeat regions are important in recombination and variation in chloroplast genomes^28^. By using the REPuter algorithm, complex repeats of *Ilex* were analysed. The length of repeats is 30–66 bp, which falls into the typical range of other angiosperms^29,30^. There are only slight variations in the total number of repeats, which ranged from 19 to 26, but the number of each repeating motif is different. The most abundant repeats are direct repeats, while palindromic repeats are secondary. Complementary repeats can be observed in only eight *Ilex* species, and reverse repeats can be seen only in five *Ilex* species (Fig. 3). The variation in the repeating motifs of SSRs and complex repeats can be used as DNA markers to identify these species.

### Interspecies comparison of chloroplast genomes

**Figure 4:**
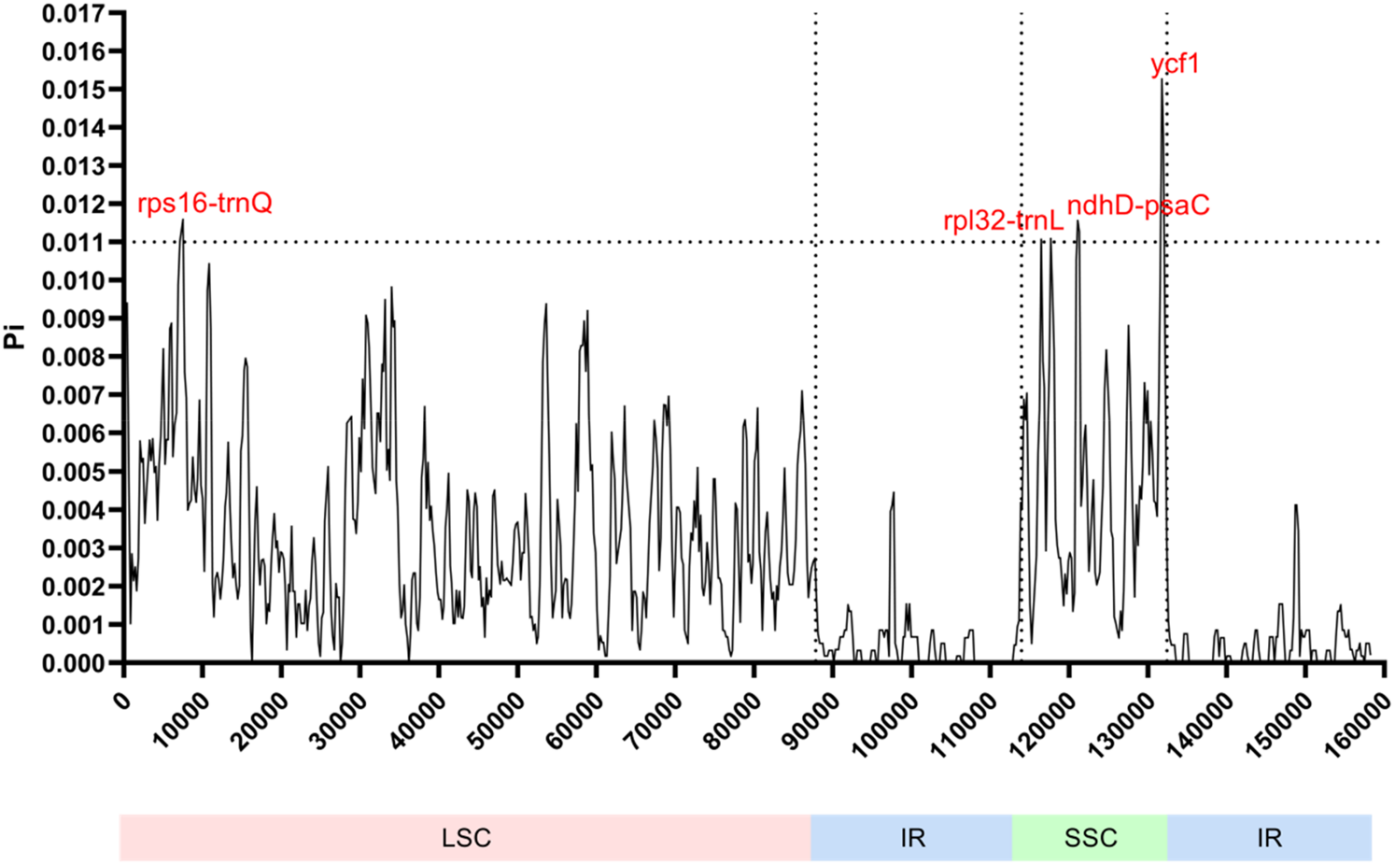
Nucleotide diversity values between 19 *Ilex* species determined by using whole chloroplast genomes. Variation hotspots (Pi > 0.011) are labelled above the corresponding gene position.

Single-nucleotide polymorphisms (SNPs) and DNA insertion-deletion mutations (indels) are important for discovering DNA markers and barcodes in medicinal herbs^31,32^. Therefore, the number of SNPs and indels were calculated by pair-wise alignment of the 19 *Ilex* chloroplast genomes. In brief, the 19 *Ilex* genomes were first aligned with each other. The number of polymorphisms was identified by an in-house developed Perl algorithm. By comparing the numbers, several groupings could be seen among the *Ilex* species. Most of the *Ilex* species have 200 to 600 SNPs and 60 to 150 indels. The average numbers of SNPs and indels are 436 and 100, respectively. On the other hand, fewer than 80 SNPs and 20 indels were discovered among *I. lohfauensis, I. championii* and *I. hanceana*. Relatively small numbers of SNPs and indels were also observed in the group of *I. kwangtungensis, I. dasyphylla,* and *I. lancilimba* and the group of *I. latifolia, I. cornuta, I. cinerea,* and *I. graciliflora* (Supplementary Table S4)*.* In fact, the three groups mentioned were classified as sections of *Pseudoaquifolium*, *Lioprinus* and *Ilex* in *Flora of China*. The same classification was given in our phylogenetic analysis. To locate the divergent hotspots, sliding window analysis was performed. The average Pi (*π*) value among the 19 *Ilex* species was 0.00268, which indicated that there were only slight variations in the chloroplast genomes. However, we still found four highly varied hotspots (Pi > 0.01), *rps16-trnQ, rpl32-trnL, ndhD-psaC* and *ycf1* (Fig. 4). *rps16-trnQ* belongs to the LSC region, while the other three are located in the SSC region. The two IR regions showed the smallest divergence, which is consistent with the characteristics of other chloroplast genomes^33–35^.

To investigate the correlation between the SNPs/indels and the Pi distance, the suggested hotspot sequences of the 19 *Ilex* chloroplast genomes were first aligned. The numbers of SNPs and indels were then compared by using *I. latifolia* as a reference. Special features were seen in all four hotspots. First, there are 68 bp gap regions in *rps16-trnQ* of *I. triflora* and *I. viridis*. We also located 5 bp deletions in *rps16-trnQ* of *I. kwangtungensis*, *I. triflora*, *I. lanchilimba I. dasyphylla* and *I. viridis*. Second, 5 bp truncations were also discovered in *rpl32-trnL* of *I. latifolia, I. ficoidea, I. cinerea* and *I. cornuta*. All of these species were members in the *Ilex* section*.* However, as the deletions are small in size*,* the divergence distance of this region is relatively lower than that of the other suggested hotspots. Third, although the *ndhD-psaC* intergenic spacer is relatively short (approximately 130 bp) compared with that of the other suggested hotspots, 12 SNPs were located in *I. kwangtungensis*, *I. triflora*, *I. lanchilimba I. dasyphylla* and *I. viridis*. The grouping was consistent with the observation in *rps16-trnQ.* Fourth, 29 bp deletions were observed in *ycf1* of *I. asprella* var. *tapuensis* and *I. chapaensis*, which may explain the unexpected position of these two species in the phylogenetic tree. All of the observations suggested that the SNPs/indels play an important role in the divergence distance, which is potentially useful for section classification. After sliding window analysis, a sequence identity plot was also constructed by using the online program mVISTA. Similar to in Yao’s study, the complete chloroplast genome of *Helwingia himalaica* was used as the reference genome for comparison^17^. We found that the gene arrangement and contents of the *Ilex* genus genomes are similar to those of the *Helwingia* genome (Supplementary Fig. S1). Several divergence hotspots, *trnK-rps16-trnQ, ndhF-rpl32-trnL* and *ycf1,* were identified, and the IR regions are mostly conserved. On the other hand, groupings in the gene arrangement could also be observed in the sequence identity plot. Compared with the other *Ilex* genomes, truncation of *rpoB-trnC* was observed in *I. lohfauensis, I. hanceana* and *I. championii.* Truncation of *ycf4-cemA* was also discovered in the group of *I. latifolia, I. ficoidea, I. cinerea, I. graciliflora* and *I. cornuta*. As expected, the groupings were similar to our findings in the SNP investigation and phylogenetic analysis, which suggested that the deletions in *rpoB-trnC* and *ycf4-cemA* may act as DNA markers in the *Hanceanae* series and the *Ilex* section, respectively.

### Genetic interspecies divergence and intraspecific variation

To validate the reliability of the suggested variation hotspots, interspecific divergence and intraspecific variation of these regions were compared. In brief, all 19 Hong Kong *Ilex* plastomes were aligned. Interspecies divergence (*π*) of the hotspots was then compared by DnaSP (Supplementary Table S5). In the aspect of intraspecific comparison, the published reference genomes of *Ilex* were first retrieved from public resources. They were then aligned with our corresponding *Ilex* plastomes (MT704834, MT764239, and MT764252), and their numbers of SNPs and indels were compared (Table 3). Among the four suggested hotspots, the *ndhD-psaC* intergenic spacer showed the highest interspecific distance. The interspecific distances of *rps16-trnQ* and *rpl32-trnL* were comparable. The divergence of *ycf1* was lower than expected, as the upstream sequences of *ycf1* were mostly conserved. Variations were commonly seen only at the 3′ end since the 3′ end overlapped with the SSC/IRb junction. All of the suggested hotspots had a higher divergence than the interspecific distance of the whole cp genome. Moreover, compared with that of the available published genomes, the intraspecific variation of the complete plastomes was also extremely low. There are 10 SNPs in the plastomes of *I. asprella* and *I. cornuta*. There are also 2 and 14 indels in these two species, respectively. Only one SNP and no indel were observed in the plastomes of *I. latifolia*. We showed that the chloroplast genomes are highly conserved within the same species, and four suggested hotspots, *rps16-trnQ, rpl32-trnL, ndhD-psaC* and *ycf1*, can potentially be used for species classification.

**Table 3:** Intraspecific comparison of the single-nucleotide polymorphisms (SNPs) and indels of Hong Kong *Ilex* and reference genomes.

Compared species	Number of SNPs	Number of indels
*Ilex asprella* (NC045274 & MT704834)	10	2
*Ilex cornuta* (NC044416 & MT764252)^18^	10	14
*Ilex latifolia* (NC047291 & MT764239)^46^	1	0

### IR expansion and contraction investigation

**Figure 5:**
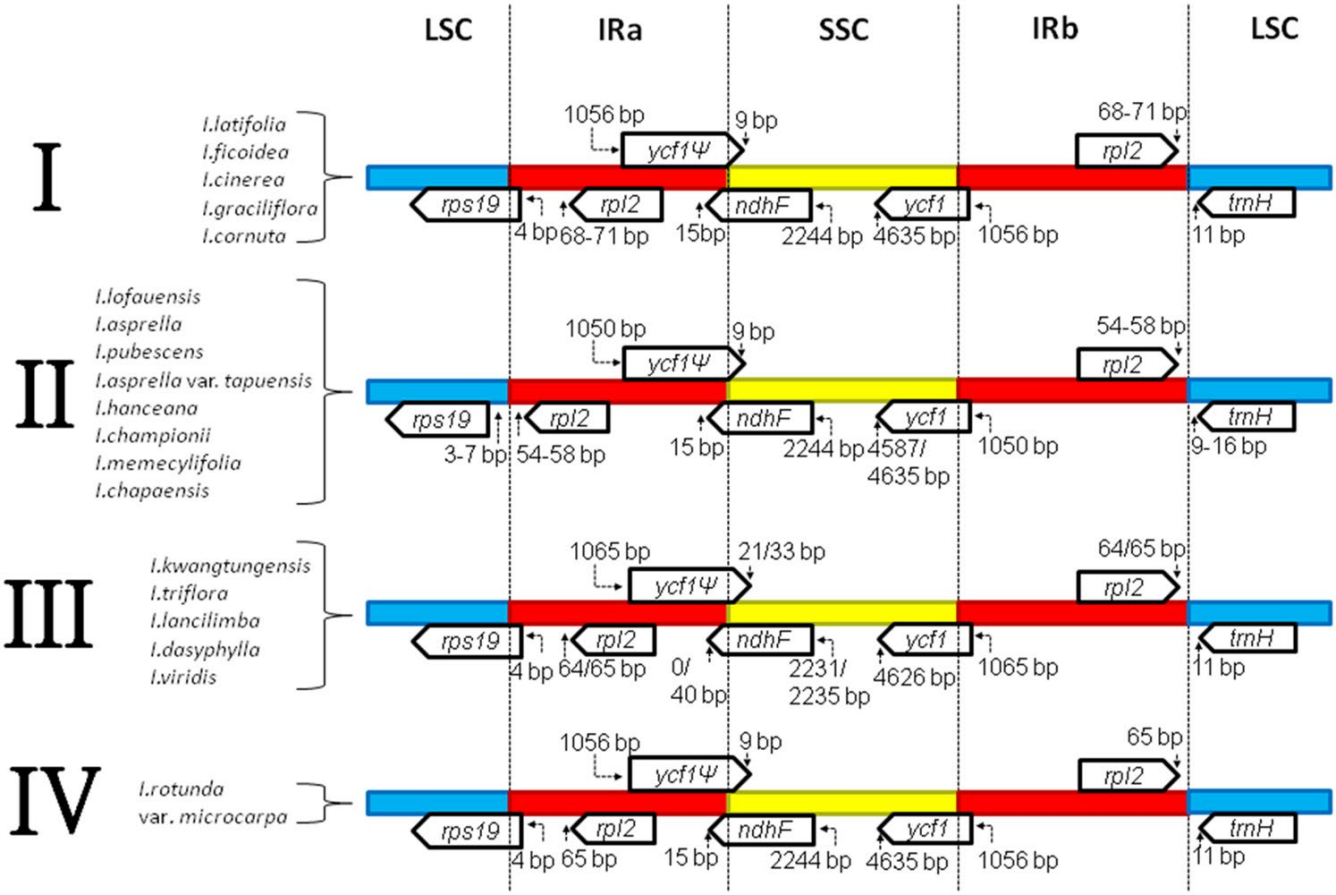
Comparison of the junction regions among 19 Hong Kong *Ilex* species. *Ψ* indicates pseudogenes. Slashes indicate the location on the genomes. Numbers above the genes denote the distance between the end of the gene and the border sites. The figure is not to scale.

In addition to the investigation of nucleotide divergence, expansion and contraction of the border regions were also analysed for the 19 Hong Kong *Ilex* species (Fig. 5). The gene arrangement of all *Ilex* species is highly conserved, in which *rps19, rpl2, ycf1*, *ndhF* and *trnH* are present at the junction of LSC/IRa, IRa/SSC, SSC/IRb, and IRb/LSC. Most of the junctions are stable; however, we could still classify the features into four distinct groups (groups I–IV) (Fig. 5; Supplementary Table S6). In brief, the lengths of the *ycf1* pseudogene (*ycf1*Ψ) in group I and group III *Ilex* are 1056 bp and 1065 bp, respectively. The *rps19* gene of group II *Ilex* is found within the LSC region, while it is located across the LSC/IR boundary in other groups of *Ilex*. The sizes of *rpl2* and *ndhF* also varied in these three groups of *Ilex.* Group IV *Ilex* possess features observed in both group I, group II and group III *Ilex*. On the other hand, subgrouping was observed in group II and group III *Ilex.* For instance, 4587 bp of the *ycf1* gene is located in the SSC region of *I. asprella* var. *tapuensis* and *I. chapaensis*; in contrast, 4635 bp of the *ycf1* gene of the other members in group II *Ilex* is located in the SSC region. The IR regions of *I. kwangtungensis* and *I. dasyphylla* show no expansion into the *ndhF* gene; in contrast, a 40 bp expansion was observed in the other members of group III *Ilex*. Moreover, the *ndhF* genes of these two species are 2231 bp but not 2235 bp across the SSC region.

### Phylogenetic analysis

**Figure 6:**
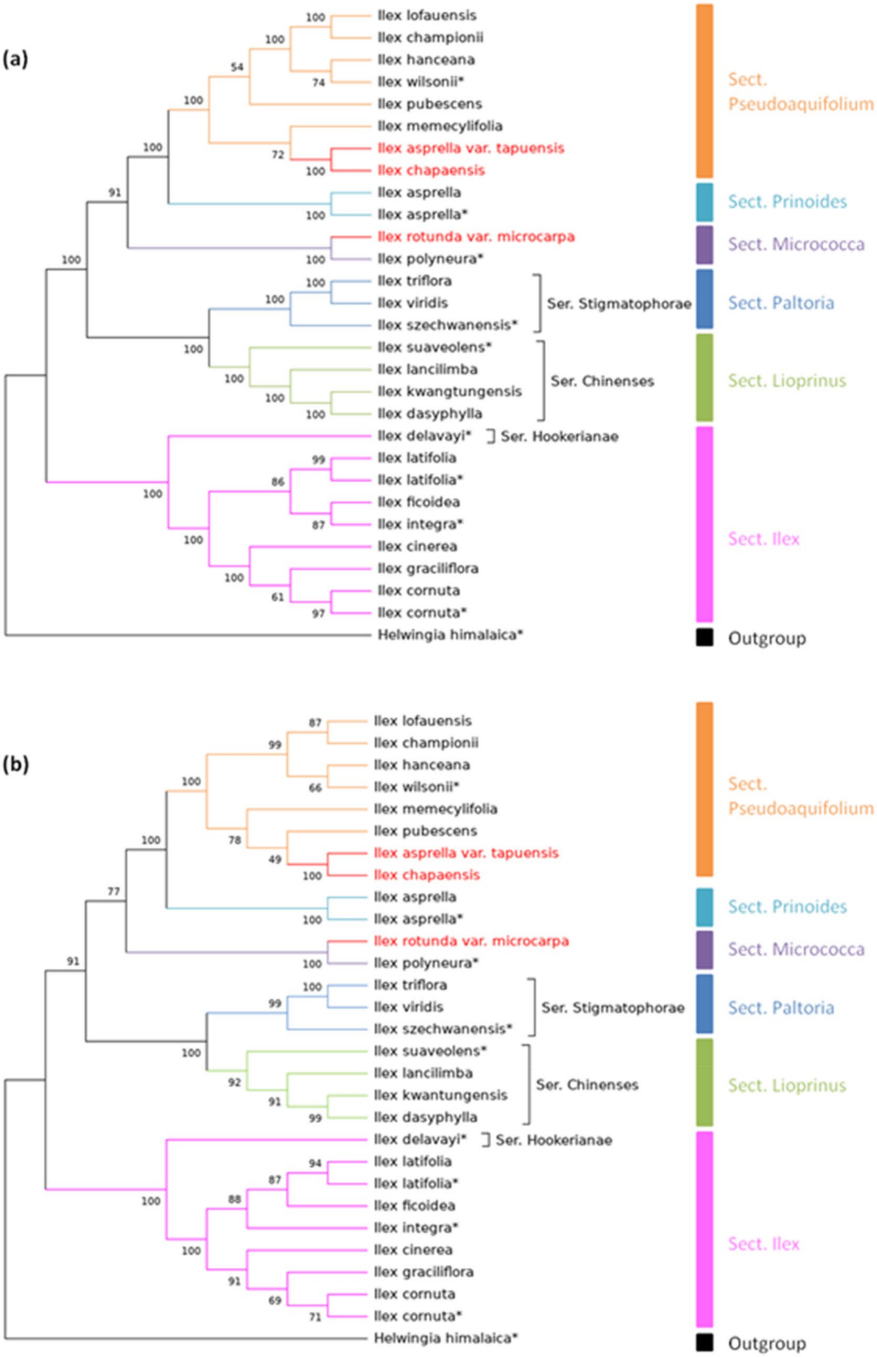
Phylogenetic tree of the *Ilex* species constructed via maximum likelihood (ML) with 1000 bootstraps by using **(a)** whole chloroplast genomes and **(b)** SSC regions. The numbers above indicate the corresponding bootstrap values, and the corresponding sections and series are labelled according to *Flora of China* and *Flora Reipublicae Popularis Sinicae*, respectively*.* Genomes obtained from previous research are marked with asterisks, while unexpected species classifications are labelled in red.

To obtain a more accurate analysis of the *Ilex* phylogeny, available *Ilex* genomes in NCBI were also included in our study. By using MEGA software, model testing was performed, and the maximum likelihood phylogenetic tree is illustrated (Fig. 6). The phylogeny matches the studies of Cuénoud and colleagues^6^. In brief, *I. triflora* and *I. viridis* were grouped together and put into the North American clade, while *I. pubescens*, *I. wilsonii*, *I. asprella* and *I. rotunda* var. *microcarpa* were assigned to the deciduous clade. *I. latifolia, I. ficoidea, I. cornuta* and *I. integra* were classified as the Eurasian clade. Similar results have been shown in research using *psbA-trnH* spacers and ITS regions^7^. Our studies were also consistent with the classification in *Flora of China* and *Flora Reipublicae Popularis Sinicae*. As expected, *I. lohfauensis, I. championii* and *I. hanceana* were grouped together, as well as *I. kwangtungensis, I. dasyphylla* and *I. lancilimba*, in which they were classified as the *Hanceanae* and *Chinensis S. Y. Hu* series, respectively. The *Hookerianae* and *Stigmatophore* series were also correctly assigned. As three out of four variation hotspots are located in the SSC region of the *Ilex* chloroplast genomes, phylogenetic studies were also carried out by using only SSC sequences. As expected, the classification was similar to the mentioned results, although some of the nodes had lower bootstrap values. All of the sections and series were assigned similarly to those with the use of the whole chloroplast genomes. The results illustrate that the SSC region, with three located DNA hotspots, can provide sufficient information for *Ilex* identification and phylogenetic investigation.

## Discussion

Here, we present the complete cp genomes of 19 native *Ilex* species in Hong Kong.

Although the organization of the chloroplast genome is highly similar among these 19 *Ilex* species and other angiosperms, significant variations in the total length, boundary of the regions and repeat distribution can be observed in the 19 plastomes (Table 1; Figs. 2, 3, 5). The genome sizes vary from 157,119 in *I. graciliflora* to 158,020 bp in *I. kwangtungensis*, which show a nearly 1 kb difference. Previous studies of seven *Ilex* chloroplast genomes found at most 300 bp differences^17^. Therefore, it is unexpected to observe more variations in our study. In many genera, including *Nicotiana* and *Quercus,* the phenomenon is due to the contraction and expansion of the IR regions^34,36^. Nonetheless, only slight variation of approximately 60 bp was observed in the *Ilex* IR regions. On the other hand, large differences were observed in the LSC regions. *I. graciliflora* and *I. kwangtungensis* possess the shortest and the longest LSC regions, respectively, with a difference of 900 bp. We then found two massive deletions in the *trnT-trnL* and *ycf4-cemA* spacers of *I. graciliflora*. The two deletions are approximately 700 bp in total. This finding may provide an explanation for the variation in genome size. In addition to the deletions in *I. graciliflora,* the *trnT-trnL* deletion was also noticed in *I. cinerea* and *I. cornuta*, while the *ycf4-cemA* deletion was observed in *I. latifolia, I. ficoidea, I. cinerea* and *I. cornuta*. These results were illustrated by mVISTA analysis (Supplementary Fig. S1). By referring to the sequence identity plot of mVISTA, a deletion in the *rpoB-trnC* spacer was found in *I. lohfauensis, I. hanceana* and *I. championii.* In fact, deletions in these regions have been widely used for species identification. The *trnT-trnL* deletion, which is 350 bp, is unique in the subfamily Cactoideae but not the other closely related cactus species^37^. Deletion in the *ycf4-cemA* intergenic region also provides a novel marker, LYCE, to discriminate between *Angelica polymorpha* and its adulterant *Ligusticum officinale*^38^. *I. lohfauensis, I. hanceana* and *I. championii* are members the *Hanceanae* series*.* Deletion in the *rpoB-trnC* intergenic region can serve as a molecular marker for species identification in these species.

All of the *Ilex* chloroplast genomes show a similar arrangement in the boundary regions (Fig. 5). In brief, SSC/IR boundaries were found within *ycf1* in all of the *Ilex* species. For this reason, the pseudogene *ycf1Ψ* was created and overlapped with *ndhF*. This phenomenon has also been found in other angiosperms, such as *Salvia* and *Prunus*^39,40^. Based on the variation of the IR expansion, we were able to divide the *Ilex* studied into four different groups. Most of the *Ilex* species in group II and group III were classified as members of the deciduous clade and the North American clade, respectively^7^. *I. latifolia, I. ficoidea,* and *I. cornuta* of the group I *Ilex* were also assigned to the Eurasian clade in Cuénoud’s study and the *Ilex* section in *Flora of China*^6,9^. Group IV *Ilex* possess features observed in group I, group II and group III *Ilex,* which may explain the unexpected classification of *I. rotunda* var. *microcarpa* in our phylogenetic studies. In addition to the general grouping of *Ilex*, subgrouping was also observed in group II and group III *Ilex* (Supplementary Table S6)*.* Compared with that in the other members of group II *Ilex*, the length of *ycf1* in *I. asprella* var. *tapuensis* and *I. chapaensis* are especially different. This result may be the reason for the unexpected position of these two species in our phylogenetic analysis. On the other hand, the boundary arrangements of *I. kwangtungensis* and *I. dasyphylla* are more similar to one another, suggesting that these two species may have a closer taxonomic relationship than the other members of group III *Ilex*. The results indicate that the phenomenon of IR expansion and the features of genome boundaries can provide useful information for *Ilex* and other angiosperm classifications.

According to our sliding window analysis, *rps16-trnQ, rpl32-trnL, ndhD-psaC* and *ycf1* show the greatest variations (Fig. 4). However, traditional barcoding regions, such as *rbcL*, *matK* and the *trnH-psbA* spacer, show only a low Pi value (Pi < 0.005). This finding illustrated that traditional DNA barcoding regions are not able to provide sufficient differences in classifying the species at a low taxonomic level. In fact, *rps16-trnQ, rpl32-trnL, ndhD-psaC* and *ycf1* are divergent hotspots in other chloroplast genomes^41^. The *rpl32-trnL* spacer is a fast-evolving sequence and is used to discriminate between *Lactuca* and *Helianthus*^42^*.* rps16-trnQ has also been suggested for DNA barcoding for 12 different genera of angiosperms^43^. *ycf1* has been proposed as the most promising cp DNA barcode, as it can distinguish the species much better than the combination of *matK, rbcL* and the *trnH-psbA* spacer^44^. As mentioned above, significant deletions were observed in *rpoB-trnC*, *trnT-trnL* and *ycf4-cemA* (Supplementary Fig. S1). These regions can also be developed for authentication purposes.

By using the complete cp genomes and SSC regions, phylogenetic analysis was performed (Fig. 6). Nearly all of the species were correctly classified in their corresponding sections, except *I. rotunda* var. *microcarpa*, *I. chapaensis* and *I. asprella* var. *tapuensis*. There are two discrepancies. First, according to *Flora Reipublicae Popularis Sinicae*, *I. rotunda* var. *microcarpa* should be in the *Lioprinus* section*.* However, *I. rotunda* var. *microcarpa* has been grouped to the *Micrococca* section in our genomic study. As the *Lioprinus* and *Micrococca* sections were classified as two different subgenera, distinct morphological variations could be observed. Some common features in the Micrococca section, such as the number of lateral veins and pyrenes, can be found in *I. rotunda* var. *microcarpa.* On the other hand, according to *Flora Reipublicae Popularis Sinicae*, the leaf surface of the *Micrococca* section is in membranous form. However, in our *I. rotunda* var. *microcarpa* specimen, the leaf surface appeared thin and leathery. In addition to *Flora Reipublicae Popularis Sinicae*, other references were considered for the phylogeny of *I. rotunda* var. *microcarpa.* Cuénoud and colleagues assigned *I. rotunda* and *I. pubescens* into the same deciduous clade, and the most closely related species with *I. rotunda* was *I. micrococca*^6^*.* The same conclusion was drawn by Manen and colleagues^7^. Interestingly, *I. micrococca* was also a member of the *Micrococca* section, which is consistent with our phylogenetic analysis. Combining these observations, we conclude that *I. rotunda* should be more closely related to the *Micrococca* section instead of the *Lioprinus* section. However, additional samples are needed to further confirm this observation. Another interesting finding was also noticed in *I. chapaensis* and *I. asprella* var. *tapuensis*. They were assigned as members of the *Pseudoaquifolium* section in our phylogenetic analysis*.* However, *Flora Reipublicae Popularis Sinicae* assigned them as members of the *Prinoides* section and grouped them together with *I. asprella*. This discrepancy needs further address by analysing nuclear and mitochondrial markers.

## Materials and methods

### Plant materials

Fresh young leaves of *Ilex* plants were collected in Hong Kong, China. Species were identified by Dr. David T.W. LAU (Curator of Shiu-Ying Hu Herbarium, School of Life Sciences, CUHK), with *Flora of Hong Kong* and *Flora of China.* Specimens with vouchers listed in Supplementary Table S1 were deposited in Shiu-Ying Hu Herbarium, School of Life Sciences, the Chinese University of Hong Kong. All *Ilex* species are not controlled under Protection of Endangered Species of Animals and Plants Ordinance (Cap. 586), Forests and Countryside Ordinance (Cap. 96), and not listed as rare and precious plants of Hong Kong. Their status are shown in Agriculture, Fisheries and Conservation Department (AFCD) Herbarium webpage (https://www.herbarium.gov.hk/result_list.aspx). Moreover, all of the individuals are collected with AFCD staff. All collections are permitted and legal.

### DNA extraction and chloroplast genome sequencing

Fresh *Ilex* leaves (100 mg) were used to extract total genomic DNA by using a DNeasy Plant Pro Kit (Qiagen Co., Hilden, Germany) following the manufacturer’s protocol. Extracted DNA was quantified in a NanoDrop Lite (Thermo Fisher Scientific, Massachusetts, USA; quality cut-off, OD 260/280 ratio between 1.7 – 2.0) and Qubit 2.0 (Invitrogen, Carlsbad, USA). DNA was visualized by 1% agarose gel electrophoresis for quality checks. Illumina 150 bp paired-end (PE) libraries for each *Ilex* species were constructed and sequenced on the NovaSeq 6000 platform (Illumina Inc., San Diego, CA, USA) by Novogene Bioinformatics Technology Co., Ltd. (https://en.novogene.com/, Beijing, China). Poor-quality reads (Phred score < 33) were removed by quality trimming in the CLC Assembly Cell package v5.1.1 (CLC Inc., Denmark).

### Genome assembly and annotation

Clean filtered reads were assembled into contigs using the CLC de novo assembler in CLC Assembly Cell package and SOAPdenovo v3.23 with default parameters. Gaps were filled by the Gapcloser module in SOAP package. Contigs were then aligned to the reference genome, *Ilex cornuta* (NC044416), and assembled into a complete chloroplast genome. Genome annotation was performed on the GeSeq platform by using complete cp genomes of *Ilex cornuta* (NC044416) and *Ilex integra* (NC044417) as references. A few adjustments for protein-coding genes and start and stop codons were performed manually. Chloroplast circular maps were then drawn in OGDRAW v1.3.1 (http://ogdraw.mpimp-golm.mpg.de/) according to the adjusted genome annotation^45^. All of the annotated genomes were deposited in GenBank with the accession numbers listed in Table 1.

### Repeat sequence identification

Repeat motifs in *Ilex* species were identified by two different programs. Regarding microsatellites, the positions and motifs of simple sequence repeats (SSRs) were analysed by MISA software (https://webblast.ipk-gatersleben.de/misa/). To screen for the SSRs, they were identified with thresholds of 10, 5, 4, 3, 3, and 3 repeat units for mono-, di-, tri-, tetra-, penta-, and hexa-nucleotides, respectively. To identify the long repeat motifs, REPuter v1.0 was used to locate forward, reverse, complementary and palindromic sequences, with a minimum repeat size of 30 bp and 90% identity. Statistical analysis was performed by GraphPad Prism v9.0.0 (GraphPad Software, La Jolla, CA, USA).

### Chloroplast genome comparison

All 19 *Ilex* complete chloroplast genomes were aligned using MAFFT v7.0 and manually adjusted in BioEdit v7.2. The number and position of indels and SNPs were then investigated by an in-house python code developed by Seoul National University. Sliding window analysis was also performed to compare the nucleotide divergence (*π*) among the 19 *Ilex* species using DnaSP v6.0. The window length was set as 600 bp with a 200 bp step size. To illustrate the interspecific variations, full alignments of the complete chloroplast genomes of all 19 *Ilex* species and *Helwingia himalaica* (NC031370) were visualized in the mVISTA program under Shuffle-LAGAN mode.

### Phylogenetic analysis

Complete chloroplast genomes and SSC sequences of the 19 *Ilex* species together with those of *Ilex* species available in NCBI, which were *Ilex asprella* (NC045274), *Ilex cornuta* (NC044416), *Ilex integra* (NC044417)*, Ilex latifolia* (NC047291), *Ilex szechwanensis* (KX426466)*, Ilex suaveolens* (MN830249), *Ilex polyneura* (KX426468), *Ilex delavayi* (KX426470) and *Ilex wilsonii* (KX426471), were used for phylogenetic analysis. The chloroplast sequence of *Helwingia himalaica* (NC031370) was also included as an outgroup. All of the chloroplast sequences were then aligned with MAFFT v7.0. The best nucleotide substitution model (GTR + R + I) was tested. Maximum likelihood (ML) with 1000 bootstrap replicates was constructed with MEGA-X software. Most of the taxonomic sections were labelled according to *Flora of China*. The *Micrococca Prinoides* sections and taxonomic series were labelled according to *Flora Reipublicae Popularis Sinicae.*

## Conclusion

The chloroplast genomes of 19 *Ilex* species were sequenced, among which 14 genomes were reported for the first time in this study. Variations in the repeating motifs were illustrated, and four divergence hotspots were located. These can be potentially useful to develop authentication markers for medicinal *Ilex*. Together with other published *Ilex* chloroplast genomes, the phylogenetic relationship of 25 *Ilex* plastomes was studied. This work gives us a better understanding of *Ilex* phylogeny.

## Supplementary Information

Supplementary Information 1.

Supplementary Information 2.

Supplementary Information 3.

Supplementary Information 4.

Supplementary Information 5.

Supplementary Information 6.

Supplementary Information 7.

## Data Availability

The complete chloroplast sequences generated and analysed during the current study are available in GenBank (MT704834, MT764239-MT764254 and MT767004-MT767005).
