# Supplementary material for: Comparative analysis and phylogenetic investigation of Hong Kong Ilex chloroplast genomes
Source: Sci Rep. 2021 Mar 4;11:5153. doi: 10.1038/s41598-021-84705-9 (PMC7933167; doi:10.1038/s41598-021-84705-9)
Supplement: Supplementary file 7 — Supplementary Information 7. [file 41598_2021_84705_MOESM7_ESM.pdf]

(a)

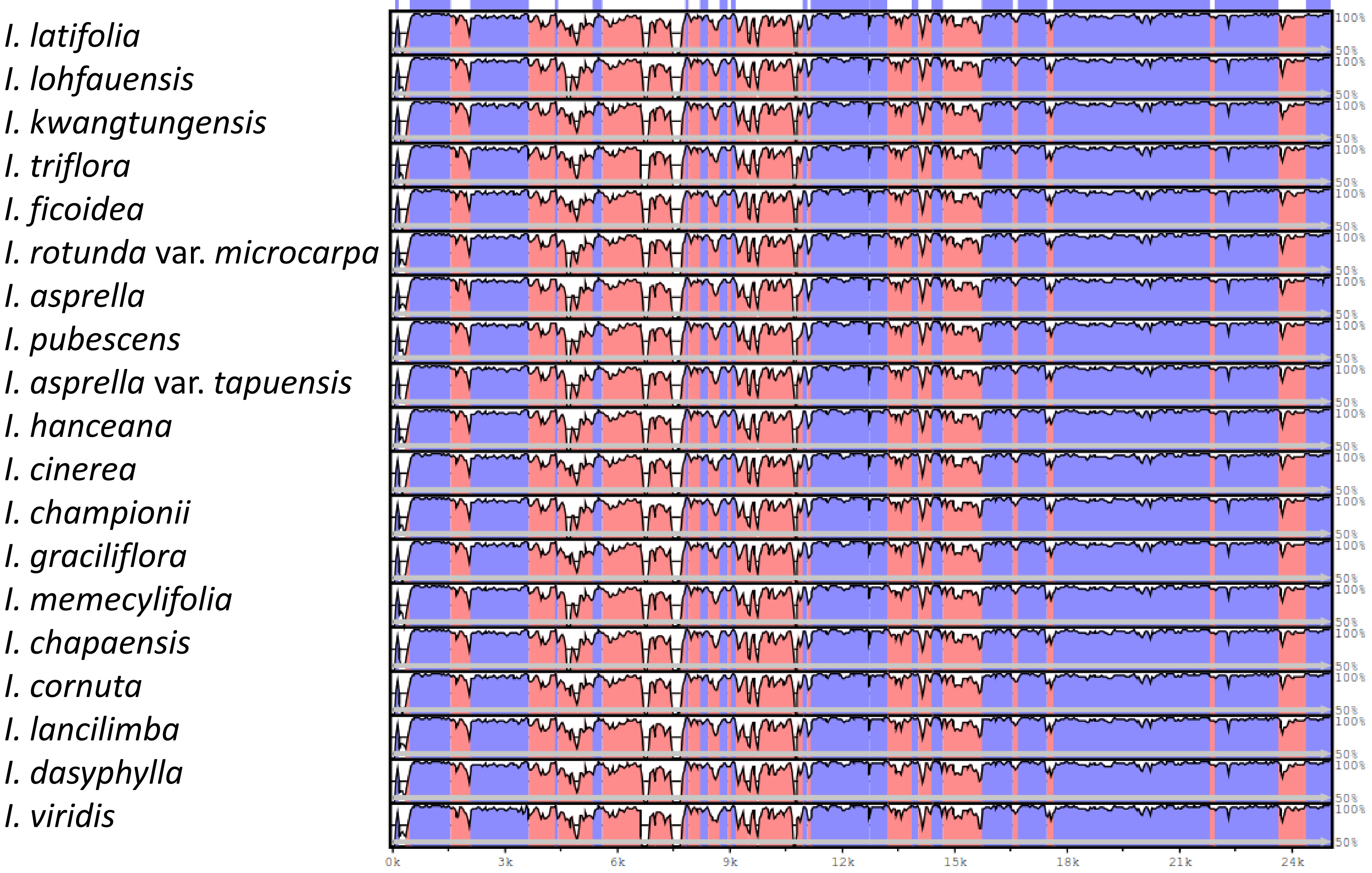

I. latifolia
I. lohfauensis
I. kwangtungensis
I. triflora
I. ficoidea
I. rotunda var. microcarpa
I. asprella
I. pubescens
I. asprella var. tapuensis
I. hanceana
I. cinerea
I. championii
I. graciliflora
I. memecylifolia
I. chapaensis
I. cornuta
I. lancilimba
I. dasyphylla
I. viridis
trnH
psbA
matK
trnK
rps16
trnQ
psbK
psbI
trnS
trnR
atpA
atpF
atpH
atpI
rps2
rpoC2
rpoC1
rpoB
100%
50%
0k
3k
6k
9k
12k
15k
18k
21k
24k

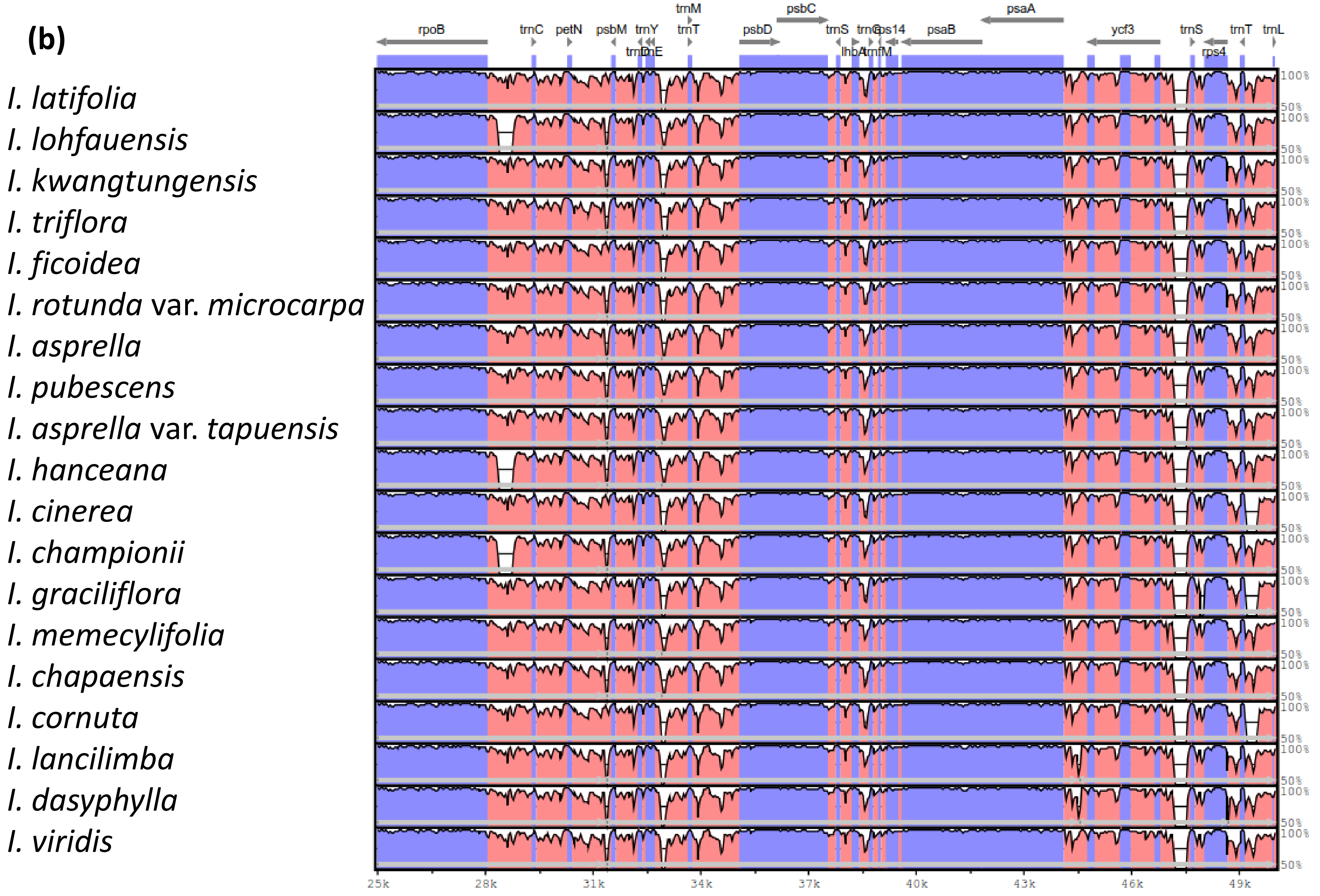

(b)
I. latifolia
I. lohfauensis
I. kwangtungensis
I. triflora
I. ficoidea
I. rotunda var. microcarpa
I. asprella
I. pubescens
I. asprella var. tapuensis
I. hanceana
I. cinerea
I. championii
I. graciliflora
I. memecylifolia
I. chapaensis
I. cornuta
I. lancilimba
I. dasyphylla
I. viridis
rpoB
trnC
petN
psbM
trnY
trnM
trnT
psbD
psbC
trnS
psaB
psaA
ycf3
trnS
rps4
trnT
trnL
100%
50%
25k
28k
31k
34k
37k
40k
43k
46k
49k

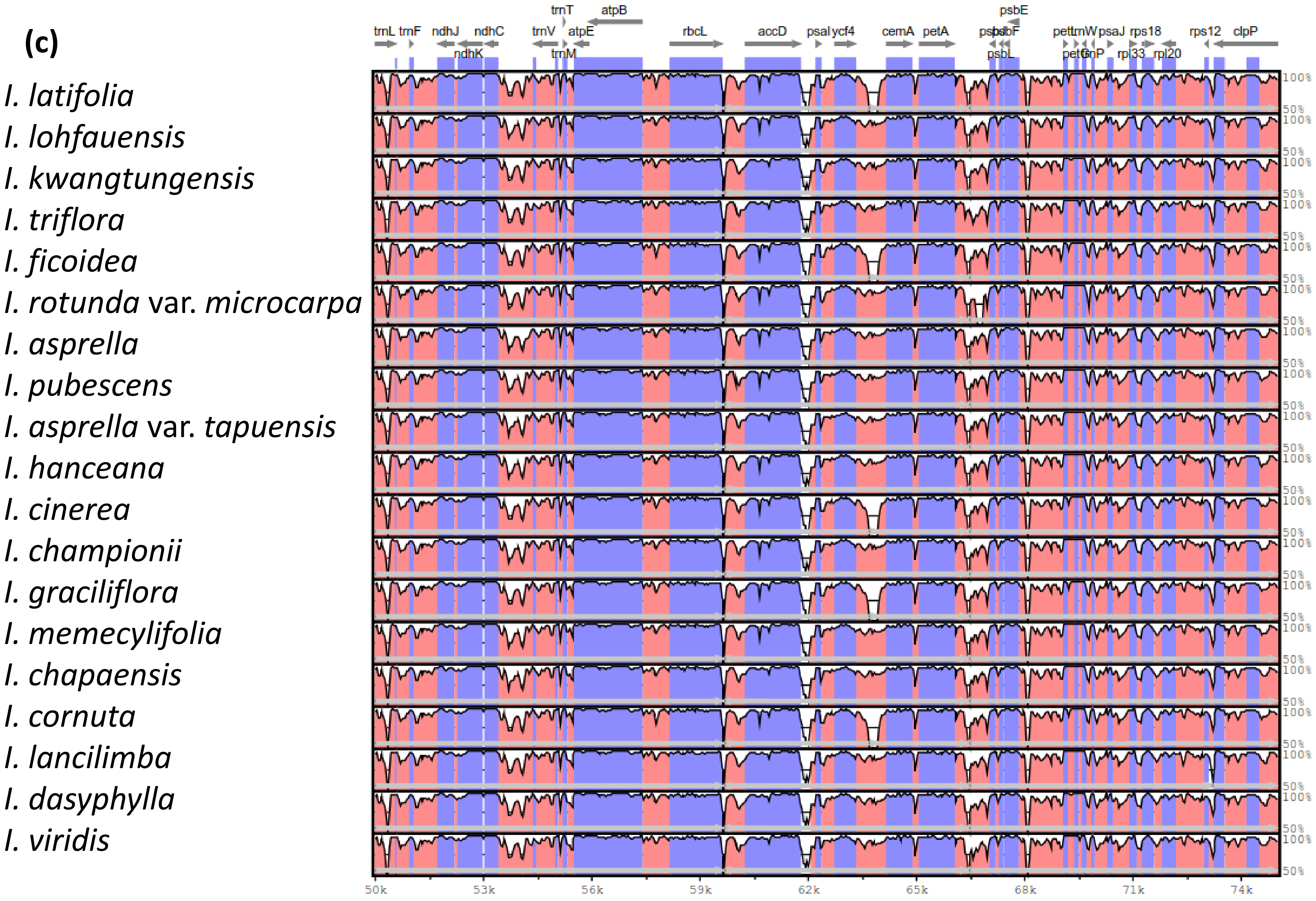

(c)
trnT
atpB
psbE
trnL
trnF
ndhJ
ndhC
trnV
atpE
rbcL
accD
psaI
ycf4
cemA
petA
psbF
petL
trnW
psaJ
rps18
rps12
clpP
ndhK
trnM
psbL
petG
trnP
rpl33
rpl20
I. latifolia
I. lohfauensis
I. kwangtungensis
I. triflora
I. ficoidea
I. rotunda var. microcarpa
I. asprella
I. pubescens
I. asprella var. tapuensis
I. hanceana
I. cinerea
I. championii
I. graciliflora
I. memecylifolia
I. chapaensis
I. cornuta
I. lancilimba
I. dasyphylla
I. viridis
100%
50%
50k
53k
56k
59k
62k
65k
68k
71k
74k

(d)

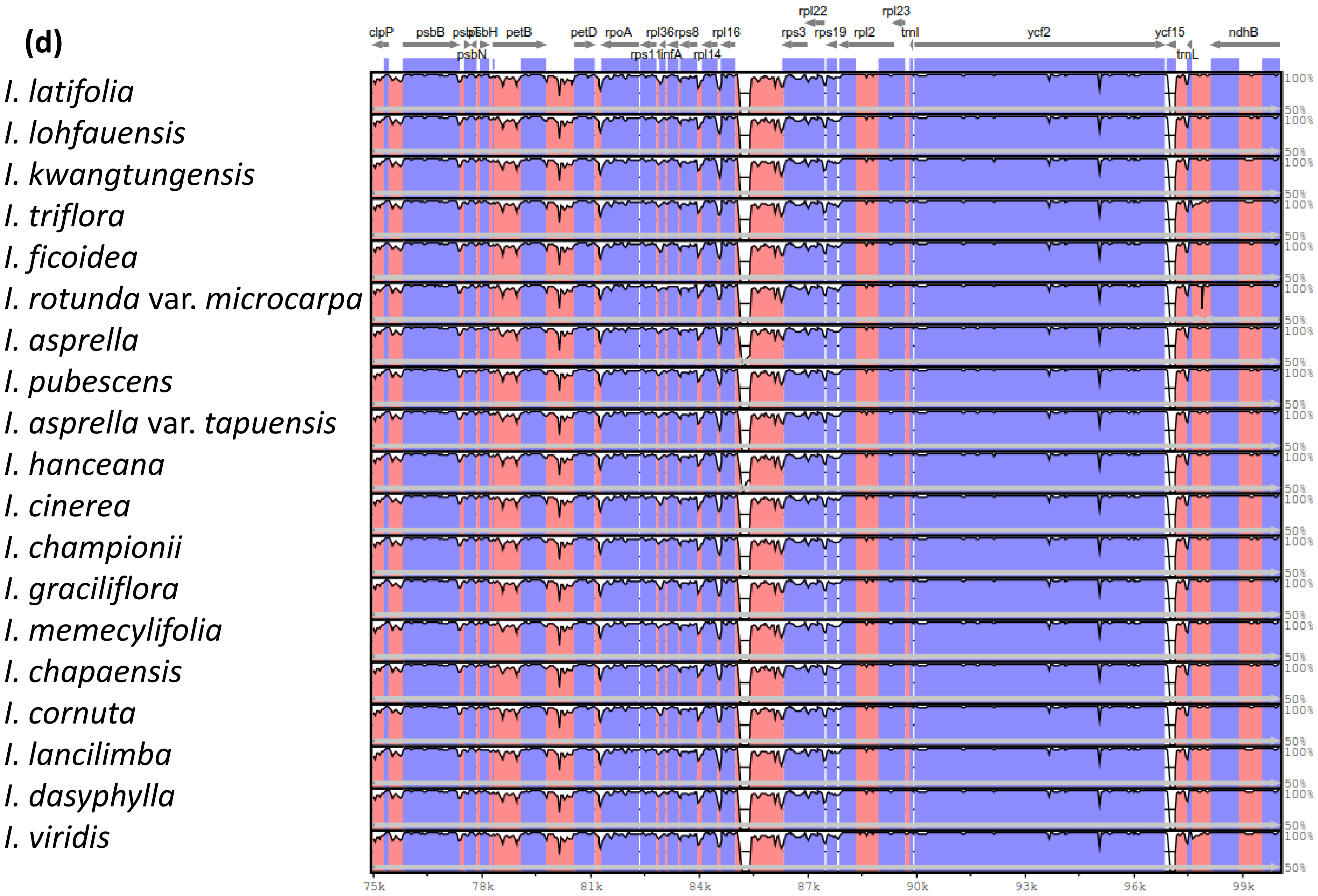
I. latifolia
I. lohfauensis
I. kwangtungensis
I. triflora
I. ficoidea
I. rotunda var. microcarpa
I. asprella
I. pubescens
I. asprella var. tapuensis
I. hanceana
I. cinerea
I. championii
I. graciliflora
I. memecylifolia
I. chapaensis
I. cornuta
I. lancilimba
I. dasyphylla
I. viridis
rpl22
rpl23
clpP
psbB
psbT
psbH
petB
petD
rpoA
rpl36
rps8
rpl16
rps3
rps19
rpl2
trnI
ycf2
ycf15
ndhB
psbN
rps11
infA
rpl14
trnL
100%
50%
75k
78k
81k
84k
87k
90k
93k
96k
99k

(e)

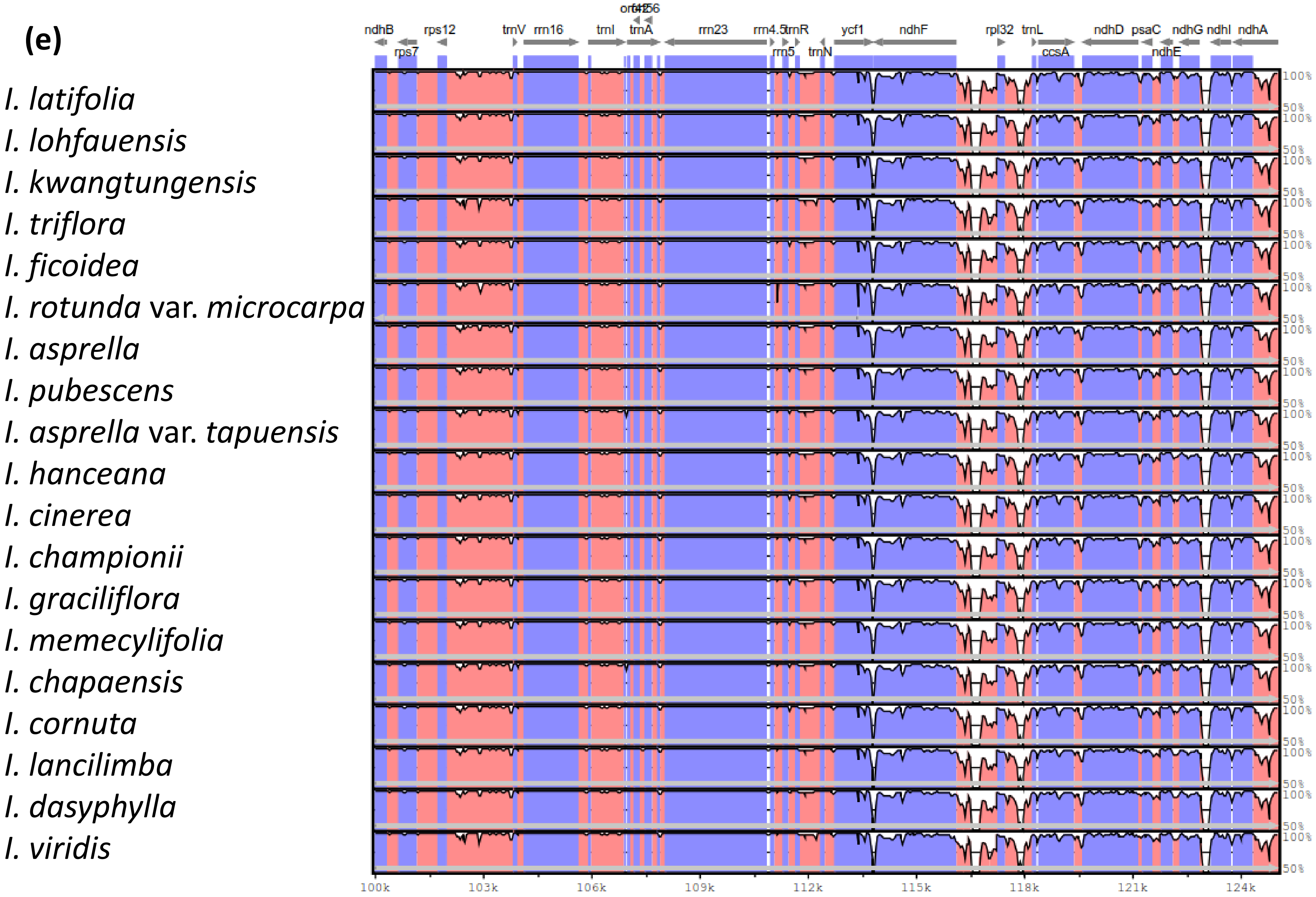
I. latifolia
I. lohfauensis
I. kwangtungensis
I. triflora
I. ficoidea
I. rotunda var. microcarpa
I. asprella
I. pubescens
I. asprella var. tapuensis
I. hanceana
I. cinerea
I. championii
I. graciliflora
I. memecylifolia
I. chapaensis
I. cornuta
I. lancilimba
I. dasyphylla
I. viridis
ndhB
rps7
rps12
trnV
rrn16
trnI
trnA
rrn23
rrn4.5
rrn5
trnR
trnN
ycf1
ndhF
rpl32
trnL
ccsA
ndhD
psaC
ndhE
ndhG
ndhI
ndhA
100%
50%
100k
103k
106k
109k
112k
115k
118k
121k
124k

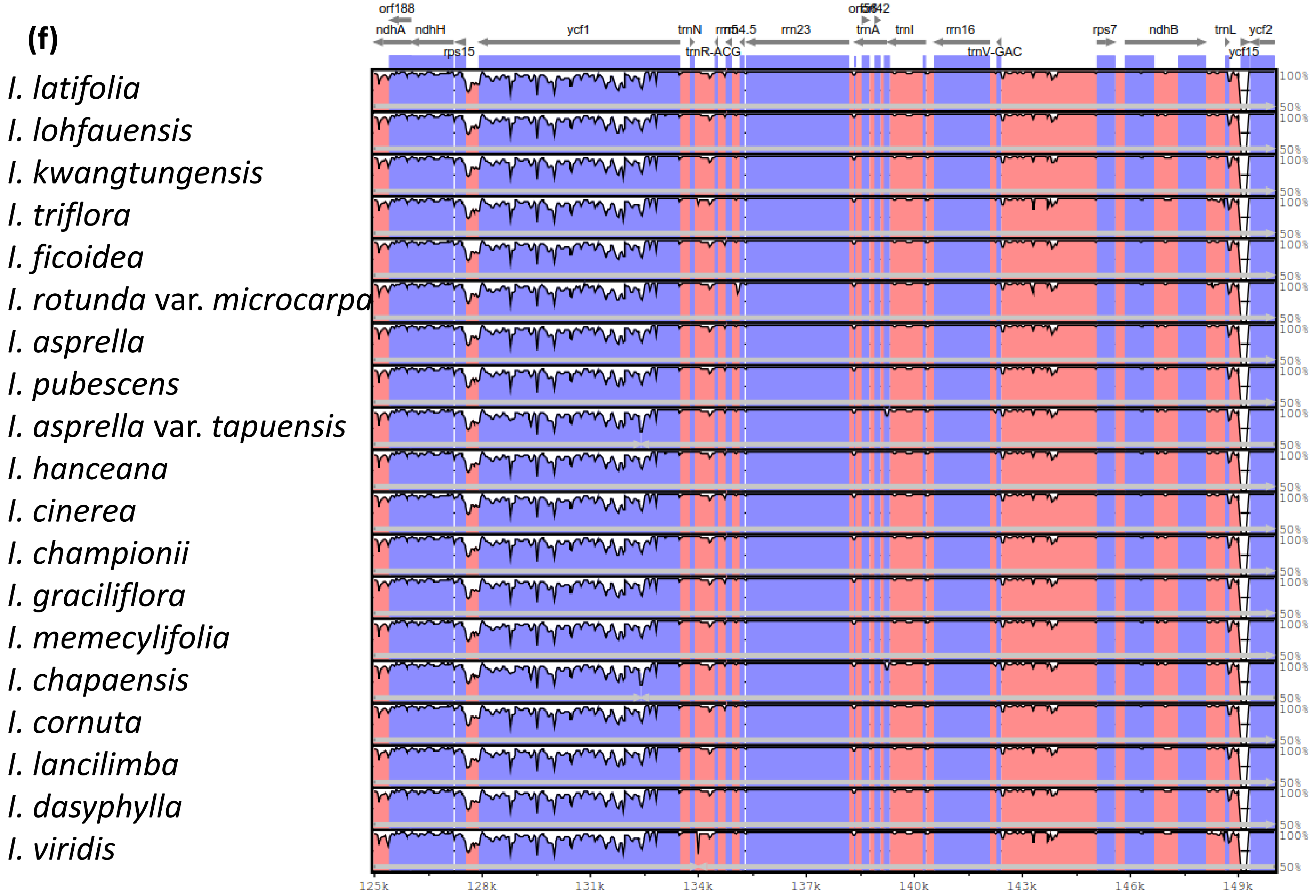

(f)
I. latifolia
I. lohfauensis
I. kwangtungensis
I. triflora
I. ficoidea
I. rotunda var. microcarpa
I. asprella
I. pubescens
I. asprella var. tapuensis
I. hanceana
I. cinerea
I. championii
I. graciliflora
I. memecylifolia
I. chapaensis
I. cornuta
I. lancilimba
I. dasyphylla
I. viridis
orf188
ndhA
ndhH
rps15
ycf1
trnN
trnR-ACG
rrn23
trnA
trnI
rrn16
trnV-GAC
rps7
ndhB
trnL
ycf2
ycf15
100%
50%
125k
128k
131k
134k
137k
140k
143k
146k
149k

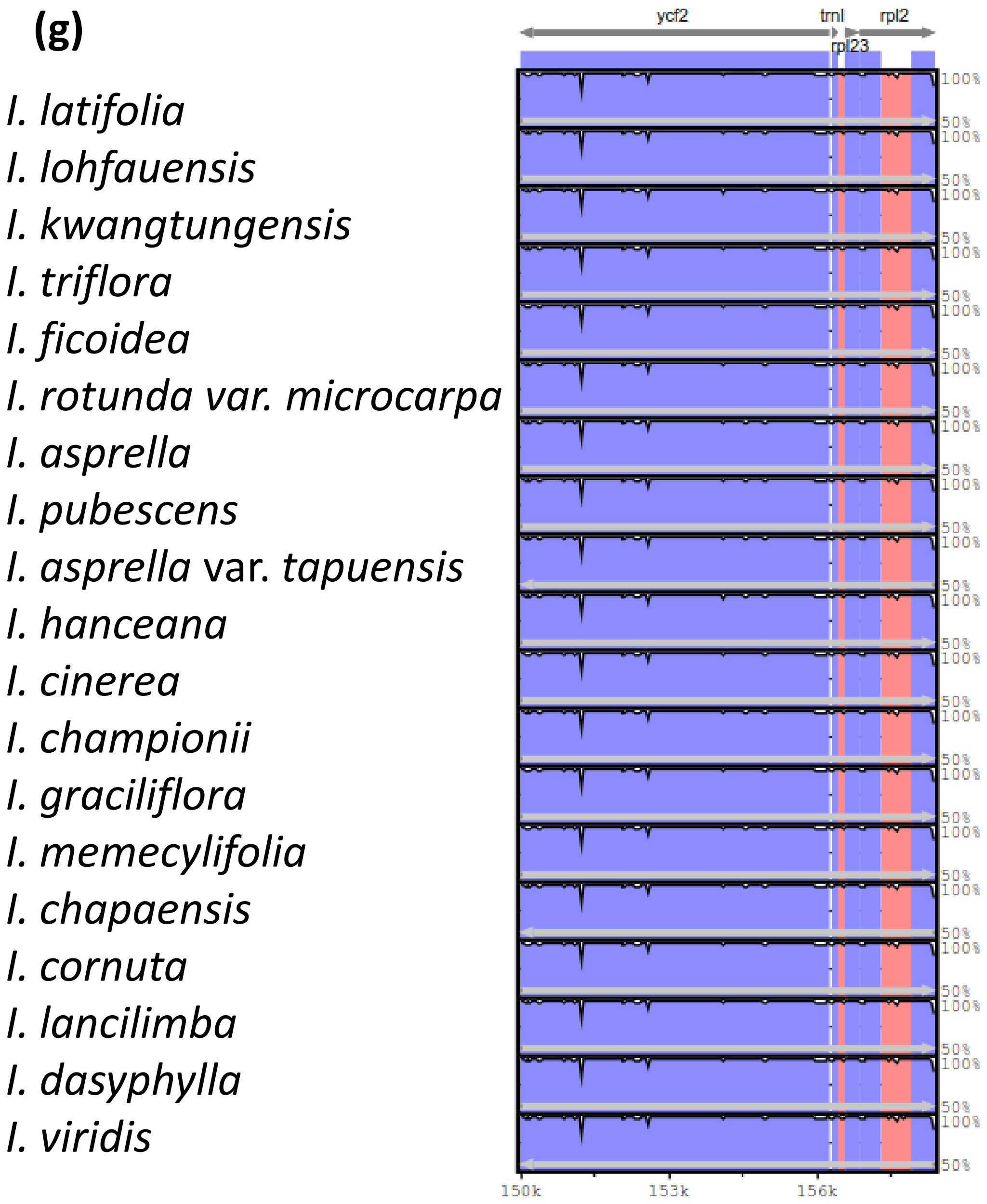
(g)
ycf2
trnI
rpl2
rpl23
I. latifolia
I. lohfauensis
I. kwangtungensis
I. triflora
I. ficoidea
I. rotunda var. microcarpa
I. asprella
I. pubescens
I. asprella var. tapuensis
I. hanceana
I. cinerea
I. championii
I. graciliflora
I. memecylifolia
I. chapaensis
I. cornuta
I. lancilimba
I. dasyphylla
I. viridis
100%
50%
150k
153k
156k

Supplementary Figure 1. Identity plot comparing the chloroplast genomes of 19 Ilex using Helwingia himalaica as a reference sequence. The vertical scale indicates the percentage of identity, ranging from 50 to 100%. The horizontal axis indicates the coordinates within the chloroplast genome. Protein-coding regions are labeled with blue while the conserved non-coding sequences (CNS) are labeled with red.
